# The amygdala's response to face and emotional information and potential category-specific modulation of temporal cortex as a function of emotion

**DOI:** 10.3389/fnhum.2014.00714

**Published:** 2014-09-11

**Authors:** Stuart F. White, Christopher Adalio, Zachary T. Nolan, Jiongjiong Yang, Alex Martin, James R. Blair

**Affiliations:** ^1^Section on Affective Cognitive Neuroscience, National Institute of Mental Health, National Institutes of HealthBethesda, MD, USA; ^2^Department of Psychology, University of California, BerkeleyBerkeley, CA, USA; ^3^Department of Psychology, Peking UniversityBeijing, China; ^4^Laboratory of Brain and Cognition, National Institute of Mental Health, National Institutes of HealthBethesda, MD, USA

**Keywords:** amygdala, animate, emotion, fusiform gyrus, temporal cortex

## Abstract

The amygdala has been implicated in the processing of emotion and animacy information and to be responsive to novelty. However, the way in which these functions interact is poorly understood. Subjects (*N* = 30) viewed threatening or neutral images that could be either animate (facial expressions) or inanimate (objects) in the context of a dot probe task. The amygdala showed responses to both emotional and animacy information, but no emotion by stimulus-type interaction; i.e., emotional face and object stimuli, when matched for arousal and valence, generate comparable amygdala activity relative to neutral face and object stimuli. Additionally, a habituation effect was not seen in amygdala; however, increased amygdala activity was observed for incongruent relative to congruent *negative* trials in second vs. first exposures. Furthermore, medial fusiform gyrus showed increased response to inanimate stimuli, while superior temporal sulcus showed increased response to animate stimuli. Greater functional connectivity between bilateral amygdala and medial fusiform gyrus was observed to negative vs. neutral objects, but not to fearful vs. neutral faces. The current data suggest that the amygdala is responsive to animate and emotional stimuli. Additionally, these data suggest that the interaction between the various functions of the amygdala may need to be considered simultaneously to fully understand how they interact. Moreover, they suggest category-specific modulation of medial fusiform cortex as a function of emotion.

## Introduction

Considerable work implicates the amygdala in emotional processing (LeDoux, 2012). There are data demonstrating greater amygdala responses to emotional (fearful) relative to neutral facial expressions (Murphy et al., 2003). But animal and human data also indicate that the amygdala is simply responsive to face stimuli (see Pessoa and Adolphs, 2010). The amygdala shows greater responses to animate stimuli, including faces (Gobbini et al., 2011; Yang et al., 2012), animals (Chao et al., 2002; Yang et al., 2012; Coker-Appiah et al., 2013) and inanimate objects moving in animate ways (Martin and Weisberg, 2003; Wheatley et al., 2007; Santos et al., 2010), relative to inanimate stimuli. There remains a question though regarding the extent to which the amygdala's response to emotional stimuli is limited to only *animate* emotional stimuli. One study examining BOLD responses to faces, animals and objects that were either emotional or neutral in the context of a repetition detection task reported an animacy-by-emotion interaction within the amygdala (Yang et al., 2012). The differential response to threatening faces vs. neutral faces was significantly greater than the differential response to threatening objects vs. neutral objects. Indeed, in this study, the amygdala showed no significant response to threatening relative to neutral objects. In contrast, two additional studies, one using a very similar paradigm to Yang and colleagues (Cao et al., 2014) and a second examining the differential response to approaching or receding animate or inanimate threats or neutral stimuli (Coker-Appiah et al., 2013), both reported main effects for emotion within the amygdala. All three of these studies reported increased amygdala responses to faces and other animate stimuli relative to objects. However, while the data reported by Yang et al. (2012) suggested the amygdala's response to emotional stimuli was confined to animate stimuli, those of Coker-Appiah et al. (2013) and Cao et al. (2014) both indicated the amygdala responded to emotional stimuli whether they were animate or not. Furthermore, other work found greater amygdala response to sharp relative to curved contours in a series of neutral objects (e.g., sharp cornered vs. round baking pans); the authors argue that sharp contours are more threatening than rounded ones (Bar and Neta, 2007). These data also suggest that amygdala responds to threat information independently of animacy information.

An important role of the amygdala concerns its role in emotional attention. The suggestion is that the amygdala primes representations of emotional stimuli in temporal cortex such that these are neurally represented more strongly than non-emotional stimuli. Thus, emotional stimuli are more likely to win the competition for representation and thereby become the focus of attention (Pessoa and Ungerleider, 2004; Blair et al., 2007). Emotional modulation of attention is thought to occur via direct feedback projections from the amygdala to visual processing areas, including temporal cortex (Pessoa et al., 2002; Mitchell et al., 2007). Interestingly, it has been argued that object concepts belonging to different categories are represented in partially distinct, sensory- and motor property–based neural networks (Caramazza and Shelton, 1998; Martin, 2007). For common tools, the neural circuitry includes the medial portion of the fusiform gyrus and posterior medial temporal gyrus, assumed to represent their visual form and action properties (motion and manipulation; Martin, 2007). For faces and animate objects, this circuitry includes two regions in posterior temporal cortex: the lateral portion of the fusiform gyrus, including the fusiform face area (FFA; Kanwisher and Yovel, 2006; Nguyen et al., 2013) and a region of posterior superior temporal sulcus (STS; Chao et al., 2002; Martin, 2007; Gobbini et al., 2011). These have been implicated in representing their visual form and motion, respectively (Beauchamp et al., 2003; Pelphrey et al., 2005; Beauchamp and Martin, 2007). Given differential representation of objects and faces within medial fusiform gyrus and lateral fusiform gyrus/STS respectively, one can anticipate emotional priming to occur in a category specific pattern within temporal cortex. However, this has not been formally tested.

Furthermore, the amygdala is sensitive to novel stimuli and shows rapid habituation to repeated presentation of the same stimulus (Breiter et al., 1996; Fischer et al., 2000, 2003; Wright et al., 2001). There are indications that this habituation effect is comparable for happy, fearful and neutral faces (e.g., Fischer et al., 2003) and for snakes and flowers (Balderston et al., 2013), the interaction between repetition effects, emotion and animacy in the amygdala has not to our knowledge been previously examined.

The goal of the current study is to examine the functional roles of the amygdala. A dot probe paradigm, rather than the previously used repetition detection or stimulus detection paradigms, was chosen because it provides the possibility of generating behavioral data regarding the functional impact of emotion and animacy information. Behavioral data that is interpretable on a trial-by-trial basis provides a useful context in which neural data can be interpreted.

The current study tests six predictions. First, given previous findings (Pessoa and Adolphs, 2010; Yang et al., 2012), we predicted that the amygdala would show increased responses to faces relative to objects. Second, based on previous findings (Fischer et al., 2003), we predicted that habituation effects would be seen in the amygdala regardless of emotional or animacy information. Third, we predicted that if the amygdala is responsive to emotional information irrespective of animacy (cf. Coker-Appiah et al., 2013; Cao et al., 2014), there would be comparably increased responses within the amygdala to emotional faces and objects relative to neutral faces and objects. Fourth, if the amygdala is only, or much more strongly, responsive to the emotional content of face stimuli (cf. Yang et al., 2012), then there would be a significant animacy-by-emotion interaction within the amygdala such that responding is significantly greater to fearful vs. neutral faces relative to threatening vs. neutral objects. Fifth, following previous work implicating medial fusiform cortex in preferential responding to inanimate stimuli (Beauchamp et al., 2002; Mahon et al., 2007; Gobbini et al., 2011), we predicted greater responses within medial fusiform cortex to objects relative to faces. Moreover, we predicted: (i) modulation by threat would only occur for inanimate object stimuli within this region; and (ii) this region would show differential connectivity such that there would be greater correlation in signaling between this region and the amygdala as a function of threatening relative to neutral objects than threatening relative to neutral faces. Sixth, following previous work implicating FFA and STS in preferentially responding to animate stimuli (Beauchamp et al., 2002; Mahon et al., 2007; Gobbini et al., 2011), we predicted greater responses within FFA and STS to faces relative to objects. Moreover, we predicted: (i) modulation by threat would only occur for faces within this region; and (ii) this region would show differential connectivity such that there would be greater correlation in signaling between this region and the amygdala as a function of threat relative to neutral faces than threat relative to neutral objects.

## Method

### Subjects

Thirty right-handed subjects (13 females; aged 21.1–36.7, mean age = 26.0, *SD* = 4.20) volunteered for the study and were paid for their participation. Subjects were in good physical health as confirmed by a complete physical exam, with no history of any psychiatric illness as assessed by the DSM-IV (1994) criteria based on the Structural Clinical Interview for DSM-IV Axis I disorders (SCID; First et al., 1997). All subjects gave written informed assent/consent to participate in the study, which was approved by the National Institute of Mental Health Institutional Review Board.

### Animacy attention task

**Figure 1:**
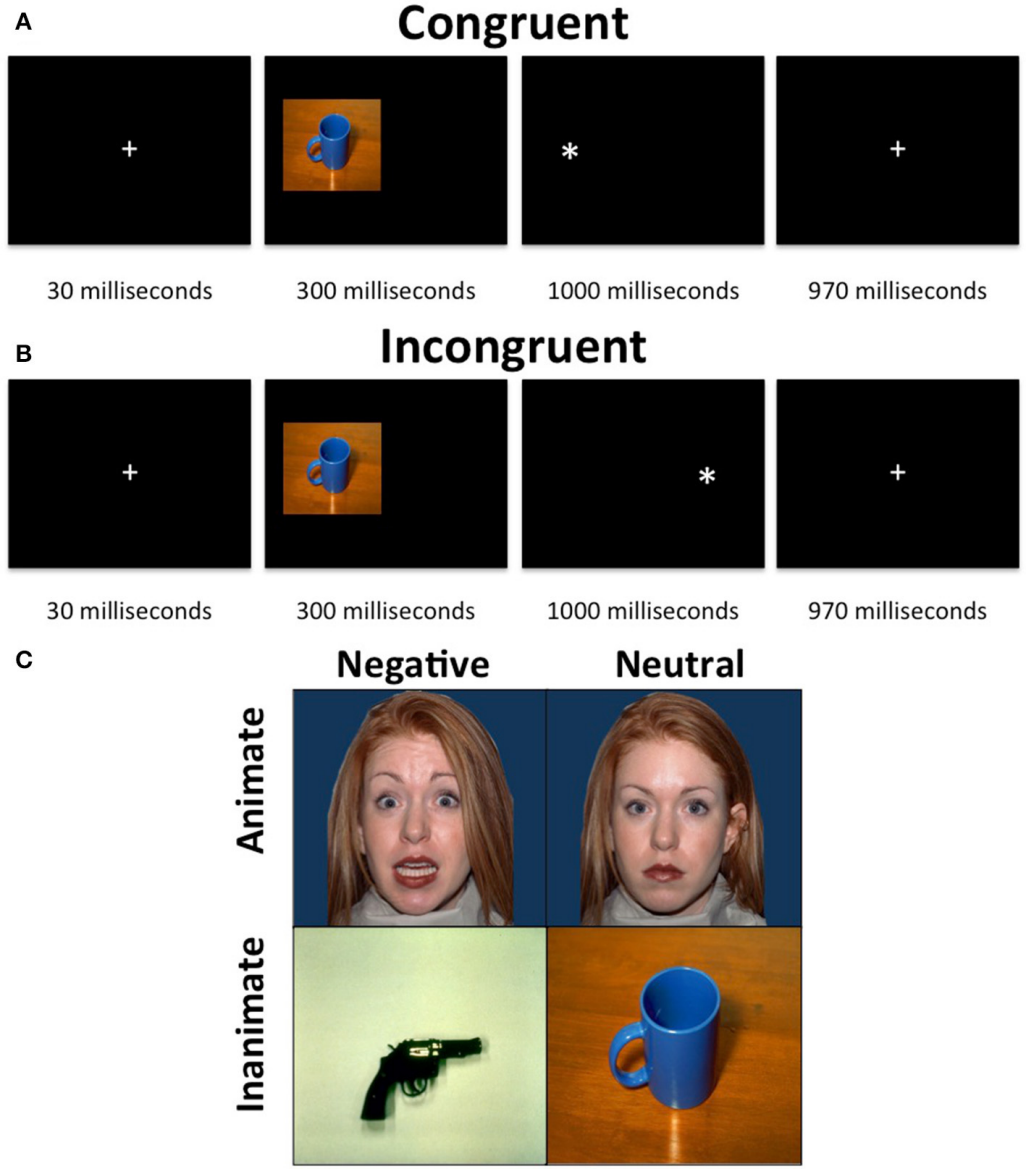
**Animacy attention task**. The task consisted of a presentation of an image, which was either neutral and animate, neutral, and inanimate, threatening and animate or threatening and inanimate, on either the left or right side of the display followed by a “^*^” probe, also on either the left or right side of the display. The participants were required to indicate, via button press, whether the probe appeared on the left or right side of the display. **(A)** Neutral inanimate congruent trial. **(B)** Neutral inanimate incongruent trial. **(C)** Example stimuli.

The animacy attention task is a dot probe task (Figure 1). The stimuli consisted of images that were threatening and animate (e.g., fearful expression), threatening and inanimate (e.g., gun), neutral and animate (e.g., neutral expressions), or neutral and inanimate (e.g., mug). There were 20 items per category (80 different images). Each image was presented two times total and never more than once per run. The stimuli were taken from the Yang et al. (2012). Based on the data from Yang and colleagues, stimuli were matched so that the facial expression stimuli did not differ from the object stimuli on valence [*t*_(78)_ = 0.938, *p* = 0.351], arousal [*t*_(78)_ = 1.632, *p* = 0.107] or luminance [*t*_(78)_ = 1.235, *p* = 0.220]. Additionally, the magnitude of these differences [(fearful faces—neutral faces)—(threatening objects—neutral objects)] was directly compared for valence and arousal. The magnitude of these differences (Cohen's d) did not significantly differ for either valence (*z* = 0.97, *p* = 0.33) or arousal (*z* = 1.51, *p* = 0.13).

Each trial began with a 30 ms fixation, followed by a 300 ms stimulus presentation on either the left of the right side of the screen occupying 40% of the width and 45% of the height of the screen. The stimuli were immediately followed by the presentation of a probe (x) for 1000 ms. During congruent trials the probe appeared on the same side of the screen as the stimulus. During incongruent trials, the probe appeared on the opposite side of the screen to the stimulus. Following the probe was a 970 ms fixation. Participants were to make a button press corresponding to the side of the screen the probe appeared on as quickly as possible after the presentation of the probe. The task included 4 runs of 2 min and 10 s each, each consisting of 10 threatening faces, 10 threatening objects, 10 neutral faces and 10 neutral objects images as well as 10 fixation trials. Sixty percent of trials were congruent and images were randomized across trials and participants. No image was presented as incongruent more than once.

### Imaging methods

#### fMRI data acquisition and preprocessing

Whole-brain blood oxygen level dependent (BOLD) fMRI data were acquired using a 3.0 Tesla GE MRI scanner. Following sagital localization, functional T2^*^ weighted images were acquired using an echo-planar single-shot gradient echo pulse sequence [matrix = 64 × 64 mm, repetition time (TR) = 2900 ms, echo time (TE) = 27 ms, field-of-view (FOV) = 240 mm (3.75 × 3.75 mm)]. Images were acquired in 34 2.5 mm axial slices with 0.5 mm spacing per brain volume. A high-resolution anatomical scan (3-dimensional spoiled gradient recalled acquisition in a steady state; *TR* = 7 ms; *TE* = 2.984 ms; 24 cm field of view; 12° flip angle; 128 axial slices; thickness, 1.2 mm; 256 × 256 matrix) in register with the EPI data set was obtained covering the whole brain.

#### Imaging data preprocessing

Data were analyzed within the framework of the general linear model using Analysis of Functional Neuroimages (AFNI; Cox, 1996). Both individual and group-level analyses were conducted. The first four volumes in each scan series, collected before equilibrium magnetization was reached, were discarded. Motion correction was performed by registering all volumes in the EPI dataset to a volume collected close to acquisition of the high-resolution anatomical dataset.

The EPI datasets for each subject were spatially smoothed (isotropic 6 mm kernel) to reduce variability among individuals and generate group maps. Next, the time series data were normalized by dividing the signal intensity of a voxel at each time point by the mean signal intensity of that voxel for each run and multiplying the result by 100, producing regression coefficients representing percent-signal change.

Following this, the following 16 regressors were generated: correct responses for the following trial types: (i) threatening faces, congruent, first exposure; (ii) threatening faces, congruent, second exposure; (iii) threatening objects, congruent, first exposure; (iv) threatening objects, congruent, second exposure; (v) neutral faces, congruent, first exposure; (vi) neutral faces, congruent, second exposure; (vii) neutral objects, congruent, first exposure; (viii) neutral objects, congruent, second exposure; (ix) threatening faces, incongruent, first exposure; (x) threatening faces, incongruent, second exposure; (xi) threatening objects, incongruent, first exposure; (xii) threatening objects, incongruent, second exposure; (xiii) neutral faces, incongruent, first exposure; (xiv) neutral faces, incongruent, second exposure; (xv) neutral objects, incongruent, first exposure; (xvi) neutral objects, incongruent, second exposure. There was also a seventeenth regressor for incorrect responses. These 17 regressors were created by convolving the train of stimulus events with a gamma-variate hemodynamic response function to account for the slow hemodynamic response. The participants' anatomical scans were individually registered to the Talairach and Tournoux atlas (Talairach and Tournoux, 1988). The individuals' functional EPI data were then registered to their Talairach anatomical scan within AFNI. Linear regression modeling was performed using the 17 regressors described above plus 6 head motion regressors. This produced a β coefficient and associated *t* statistic for each voxel and regressor.

#### fMRI data analysis

A whole-brain analysis of the BOLD data was conducted using a 2 (emotion: threatening, neutral) × 2 (object type: faces, objects) by 2 (congruency: congruent, incongruent) by 2 (exposure: first, second) ANOVA. The ClustSim program in AFNI was utilized to determine that, at an initial threshold of *p* = 0.005, a whole-brain *p* = 0.05 correction required clusters of 39 voxels. Due to its small size and theoretical importance, a small volume correction was made for the amygdala (as defined by all voxels of the Eickhoff–Zilles architectonic atlas with at least a 50% probability of being in the amygdala) at an initial threshold of 0.02, which yielded a minimum cluster size of 6 voxels. *Post-hoc* analyses were conducted on the average percent signal change taken from all voxels within each ROI generated from functional masks generated by AFNI and *t*-tests carried out in SPSS to examine interaction effects.

In addition, two generalized psychophysiological connectivity analyses was conducted to examine task-dependent connectivity between task conditions (McLaren et al., 2012). Seed regions were left and right amygdala (as defined above). For each seed region, the average activation was extracted across the time series. Interaction regressors were created by multiplying the average time series with 16 task time course vectors (one for each task condition), which were coded: 1 = task condition present and 0 = task condition not present. The average activation for the seed region was entered into a linear regression model along with the 16 interaction regressors (one per task condition), the 16 original task regressors described above, the incorrect response regressor and 6 motion regressors. A series of *t*-tests were conducted to test our hypotheses of greater connectivity between amygdala and STS and FFA for negative relative to neutral faces and greater connectivity between amygdala and medial fusiform gyrus for negative relative to neutral objects.

## Results

### Behavioral results

Two 2 (emotion: threatening, neutral) × 2 (object type: faces, objects) × 2 (congruence: congruent, incongruent) × 2 (exposure: first, second) ANOVAs were conducted on the subjects' accuracy and RT data. A significant main effect of exposure was observed for accuracy [*F*_(1, 29)_ = 6.061, *p* = 0.02]. While accuracy was high throughout the task (97.4%), participants were marginally more accurate for first [*M*_(first)_ = 0.984, *SE* = 0.005] relative to second exposures [*M*_(first)_ = 0.965, *SE* = 0.011]. No other main effects or interactions were significant.

With respect to response latency, significant main effects were observed for object type [*F*_(1, 29)_ = 8.558, *p* = 0.007], congruency [*F*_(1, 29)_ = 5.184, *p* = 0.030] and exposure [*F*_(1, 29)_ = 15.717, *p* < 0.001]. Participants were quicker to respond to faces relative to objects [*M*_(faces)_ = 413.51 (*SE* = 12.00); *M*_(objects)_ = 420.55 (*SE* = 11.25)], to congruent relative to incongruent stimuli [*M*_(congruent)_ = 412.20 (*SE* = 11.24); *M*_(incongruent)_ = 421.87 (*SE* = 12.27)] and to second exposures relative to first exposures [*M*_(first)_ = 427.07 (*SE* = 12.77); *M*_(second)_ = 406.99 (*SE* = 10.84)]. There was also an emotion-by-congruence interaction [*F*_(1, 29)_ = 5.009, *p* = 0.033]. Participants were quicker to respond to neutral congruent stimuli relative to neutral incongruent stimuli (*t* = 2.961, *p* = 0.006), but response latencies did not differ between negative congruent and negative incongruent trials (*t* = 1.150, *p* = 0.260). No other main effects or interactions were significant.

### fMRI results

A 2 (emotion: threatening, neutral) × 2 (stimulus type: faces, objects) × 2 (congruence: congruent, incongruent) × 2 (exposure: first exposure, second exposure) ANOVA was conducted on the subjects' BOLD responses (Table 1).

**Table 1:** **Brain regions demonstrating differential BOLD responses during task performance in 30 healthy participants**.

**Contrast**	**Left/right**	***BA***	**Coordinates of peak activation^a^**			***F*_(*df* = 1, 20)_**	***p***	**Voxels**
			***x***	***y***	***z***			
**MAIN EFFECT OF EMOTION**								
Amygdala	Left		−11.5	−5.5	−13.5	12.39	0.0014	8
Anterior insula/inferior frontal cortex	Right	13	40.5	13.5	17.5	15.63	<0.0001	64
Fusiform gyrus	Left	37	−37.5	−46.5	−15.5	16.64	<0.0001	42
**MAIN EFFECT OF OBJECT TYPE**								
Amygdala	Left		−14.5	−5.5	−12.5	11.58	0.0029	8
Fusiform gyrus	Left	34	−25.5	−40.5	−12.5	79.42	<0.0001	454
Fusiform gyrus	Right	34	25.5	−52.5	−12.5	82.45	<0.0001	232
Precuneus/middle occipital gyrus	Left	19	−31.5	−79.5	11.5	28.65	<0.0001	230
Precuneus/middle occipital gyrus	Right	7	28.5	−67.5	29.5	21.31	<0.0001	85
Inferior parietal cortex	Left	40	−40.5	−34.5	38.5	14.51	0.0007	52
Middle occipital/fusiform gyrus	Right	37	43.5	−58.5	−6.5	19.81	0.0001	58
Middle occipital gyrus	Right	19	37.5	−82.5	8.5	21.97	<0.0001	62
**MAIN EFFECT OF EXPOSURE**								
Postcentral gyrus/inferior parietal cortex	Left	40	−40.5	−31.5	44.5	28.11	<0.0001	522
Precentral gyrus/inferior parietal cortex	Right	6	28.5	−16.5	53.5	21.32	<0.0001	334
Middle occipital gyrus/middle temporal cortex	Right	19	46.5	−73.5	20.5	22.62	<0.0001	112
Dorsomedial frontal/anterior cingulate cortex	Left	6	−4.5	−10.5	53.5	29.59	<0.0001	68
Declive	Right	37	40.5	−61.5	−15.5	15.11	0.0005	45
Middle occipital/inferior temporal cortex	Left	37	−43.5	−64.5	−0.5	16.46	0.0003	45
**CONGRUENCE-BY-EXPOSURE INTERACTION**								
Precuneus	Right	7	13.5	−55.5	38.5	18.43	0.0002	50
**EMOTION-BY-EXPOSURE INTERACTION**								
Lingual gyrus/occipital cortex/fusiform cortex	Right	18	31.5	−70.5	−6.5	34.18	<0.0001	535
Lingual gyrus	Left	19	−31.5	−61.5	−3.5	31.82	<0.0001	108
Culmen	Left	19	−13.5	−52.5	−9.5	21.12	<0.0001	55
**EMOTION-BY-CONGRUENCE-BY-EXPOSURE**								
Inferior frontal gyrus	Left	45	−46.5	19.5	2.5	21.76	<0.0001	76
Culmen	Left	19	−7.5	−55.5	−3.5	19.49	0.0001	64
Thalamus	Left		−7.5	−7.5	8.5	20.34	<0.0001	55

a Based on the standard Talairach and Tournoux brain template, BA, Brodmann's area; df, degrees of freedom.

#### Amygdala

**Figure 2:**
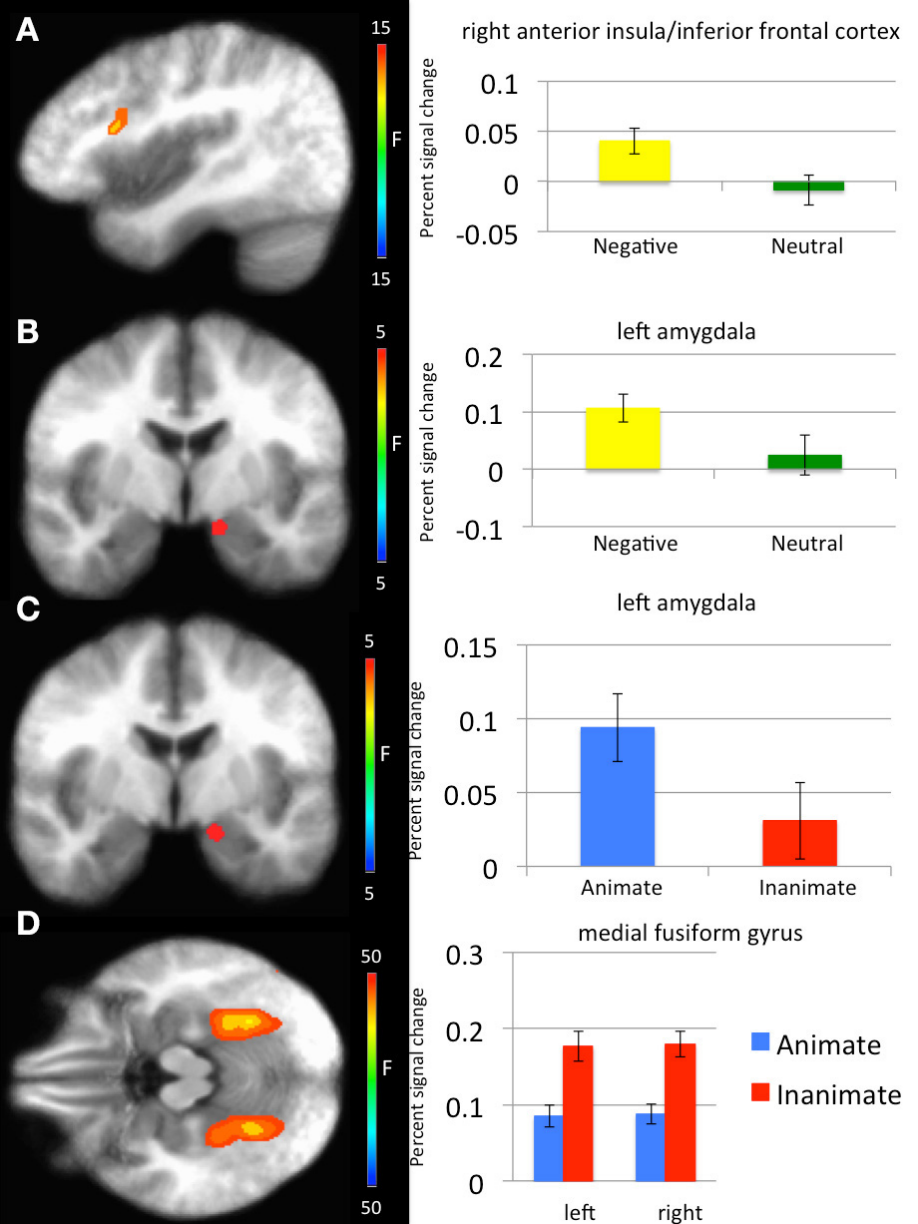
**Regions showing significant whole-brain main effects of emotion and animacy. (A)** Main effect of emotion in right anterior insula/inferior frontal cortex. **(B)** Main effect of emotion in left amygdala. **(C)** Main effect of animacy in bilateral fusiform gyrus. **(D)** Main effect of animacy in left amygdala.

With respect to our a priori predictions, the ROI analyses examining the amygdala were mixed. In line with predictions, there was a significant main effect of object type (faces > objects: left amygdala: *x*, *y*, *z* = −14.5, −5.5, −12.5, *k* = 8, Table 1, Figure 2). There was also a significant main effects of emotion (threatening > neutral: left: *x*, *y*, *z* = −11.5, −5.5, −13.5, *k* = 8). Against predictions, a main effect of exposure was not observed in amygdala.

However, several significant interactions involving exposure were observed. There was a significant emotion-by-congruence-by-exposure interaction within right amygdala. The amygdala differentiated between incongruent and congruent *negative* trials in exposure 2 (*t* = 2.448, *p* = 0.021) but not exposure 1 (*t* = 1.600, *p* = 0.120). No significant difference in activation to incongruent relative to congruent stimuli was observed for neutral stimuli (*t* < 1.072, *p* > 0.293). There was also a significant emotion-by-congruence-by-animacy-by-exposure interaction within left and right amygdala. Similar to the emotion-by-congruence-by-exposure interaction, the amygdala differentiated between incongruent and congruent *negative inanimate* trials in exposure 2 relative to exposure 1 (*t* = 3.119 and 3.536 respectively, *p* < 0.004). No other congruent vs. incongruent trial type differences for either exposure were significant (*t* < 1.432, *p* > 0.163).

#### Whole brain analysis

***Main effect of emotion***. Regions showing a significant main effect of emotion included right anterior insula cortex/inferior frontal cortex and left fusiform gyrus. Significantly greater response in both regions was observed to threatening relative to neutral stimuli (Table 1).

**Figure 3:**
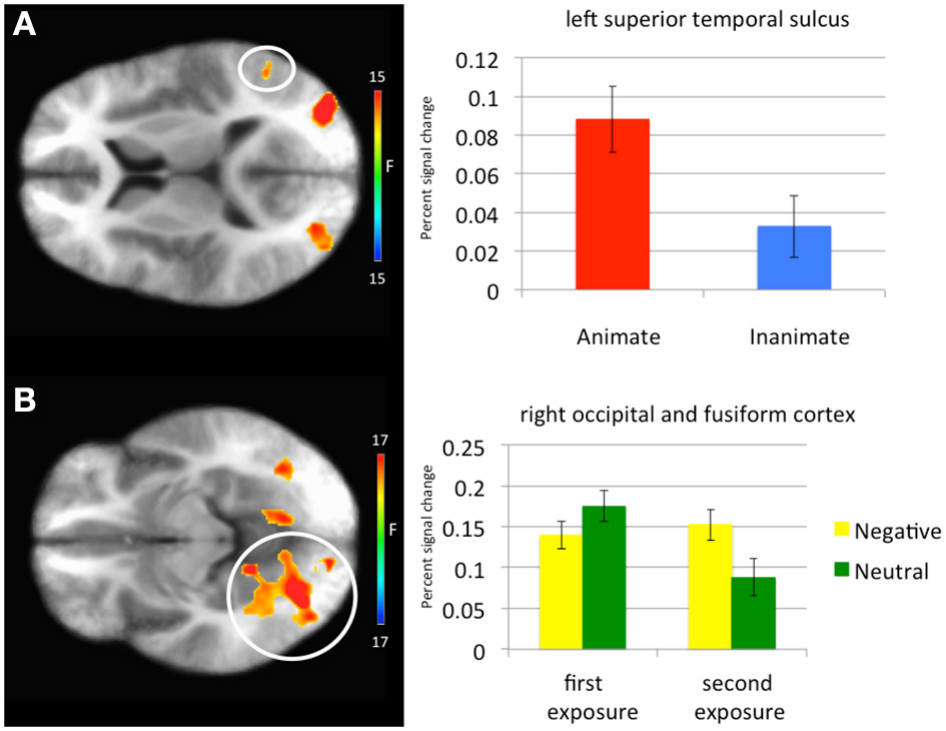
**Regions showing a significant main effect of animacy in a Superior Temporal Sulcus Region of Interest and an emotion-by-exposure interaction effect in visual cortex. (A)** Main effect of animacy in left superior temporal sulcus. **(B)** Emotion-by-exposure effect in bilateral visual cortex. The white circles specify the brain regions from which the activation in the graphs is drawn.

***Main effect of stimulus type***. Regions showing a significant effect of stimulus type included bilateral medial fusiform cortex, left inferior parietal cortex, left middle occipital cortex, two regions of right middle occipital gyrus and right precuneus. In all regions greater activation was observed to objects relative to faces (Table 1, Figure 2). Given the consistent findings in previous work suggesting that FFA and STS show increased activation to faces relative to objects, but our failure to observe this at the whole-brain level, a *post-hoc* ROI analysis was conducted. Using coordinates of peak activation from previous work examining human stimuli relative to objects, 10 mm spheres ROIs were created for STS (−47, −56, 15) and for the FFA (44, −42, −15; Beauchamp et al., 2003). At this less stringent (but still corrected for multiple comparisons; *p* = 0.005, *k* > 4 for both regions) threshold, a main effect of stimulus type was observed in STS (−49.5, −52.5, 11.5; *k* = 10; Figure 3), but not in FFA. Within STS BOLD response was greater for faces relative to objects.

***Main effect of exposure***. Regions showing a main effect of exposure included dorsomedial frontal cortex/anterior cingulate cortex (dmFC/ACC), bilateral regions encompassing motor and parietal cortex and bilateral regions of visual cortex. In all regions, greater activation was observed to the first exposure of a stimulus relative to the second exposure of the stimulus.

***Main effect of congruence***. No regions survived correction for multiple comparisons for the main effect of congruence.

***Emotion-by-exposure interaction***. Regions showing an emotion-by-exposure interaction included bilateral visual cortex and left culmen. In both regions, there was a significantly greater reduction in activity to neutral trials in exposure 2 relative to exposure 1 relative to negative trials (*t* = 4.878 and 5.529 respectively, *p* < 0.001). Indeed, there was no significant decrease in response to negative trials in exposure 2 relative to exposure 1 (*t* = 0.587 and 0.082 respectively, *p* > 0.582) (see Figure 3).

***Congruence-by-exposure interaction***. A significant congruence-by-exposure interaction was observed in right precuneus. Within this region, there was a significantly greater increase in activity for incongruent relative to congruent trials in exposure 2 [incongruent trials were associated with greater activity than congruent trials during exposure 2 (*t* = 3.126, *p* = 0.004)] relative to exposure 1 [where there was no significant difference in responsiveness to incongruent relative to congruent trials (*t* = 1.776, *p* = 0.086)] (*t* = 4.376, *p* < 0.001).

***Emotion-by-congruence-by-exposure interaction***. Regions showing an emotion-by-congruence-by-exposure interaction included left inferior frontal gyrus, left culmen and thalamus. In all regions, there was a significantly greater increase in activity for incongruent relative to congruent *negative* trials in exposure 2 (incongruent trials were indeed associated with greater activity than congruent *negative* trials during exposure 2 (*t* = 3.143–4.019, *p* = 0.004 – <0.001]) relative to exposure 1 (where there was no significant difference in responsiveness to incongruent relative to congruent negative trials [*t* = 0.517–1.846, *p* = 0.609–0.075]) (*t* = 2.076–2.915, *p* = 0.045–0.007). There was typically not a different between congruent and incongruent neutral trials for either exposure (*t* = 0.637–1.903, *p* = 0.526–0.067) (though within left culmen incongruent neutral trials were associated with greater activity than congruent neutral trials during exposure 1 [*t* = 4.035, *p* < 0.001]).

***Non-significant interactions***. No regions survived correction for multiple comparisons for the emotion-by-animacy, emotion-by-congruence, animacy-by-congruence, animacy-by-exposure, emotion-by-animacy-by-congruence, emotion-by-animacy-by-exposure, animacy-by-congruence-by-exposure and emotion-by-animacy-by-congruence-by-emotion interactions.

#### Generalized PPI analysis

**Figure 4:**
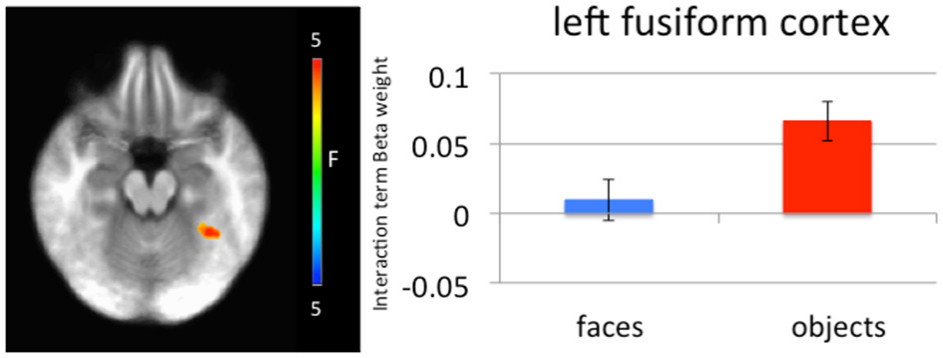
**Left medial fusiform gyrus shows a greater difference in connectivity between negative and neutral objects relative to the difference in connectivity between negative and neutral faces**. A greater difference in connectivity between right amygdala and left medial fusiform gyrus for negative compared to neutral objects was observed relative to negative compared to neutral faces.

Significantly greater connectivity between left amygdala and left medial fusiform gyrus was observed to negative relative to neutral objects (left amygdala *x*, *y*, *z* = −34.5, −46.5, −18.5, *p* = 0.005, *k* = 27; right amygdala *x*, *y*, *z* = −34.5, −46.5, −18.5, *p* = 0.005, *k* = 30; Figure 4). While these clusters did not survive correction for multiple comparison, they survived small volume correction using an anatomical mask for left fusiform cortex (*p* = 0.005, *k* > 5). This difference in connectivity with the amygdala was not observed for negative relative to neutral faces. A further *t*-test ([negative faces − negative objects] − [neutral faces − neutral objects]) found that the difference between these negative and neutral objects was significantly greater than the difference between negative and neutral faces.

## Discussion

The goal of the current study was to test contrasting assumptions regarding the responsiveness of the amygdala to emotional relative to neutral stimuli and faces relative to objects and to determine whether modulation of activity within temporal cortex was category specific. There were five main findings: First, the amygdala showed significant responses to both threatening relative to neutral stimuli (including a significant response to threatening objects relative to neutral objects) and to faces relative to objects. Second, there was no main effect of exposure within the amygdala but this primarily reflected *increased* amygdala responses to second exposure negative incongruent trials. Third, medial fusiform cortex showed significantly increased activity for objects relative to faces. Fourth, STS showed significantly increased activity for faces relative to objects, albeit at a less stringent threshold. Fifth, bilateral amygdala showed greater functional connectivity with medial fusiform cortex for threatening vs. neutral objects relative to fearful vs. neutral faces.

There have been claims that the amygdala is part of the domain-specific circuitry for responding to social and animate stimuli (Adolphs, 2009; Yang et al., 2012). In line with previous work (Gobbini et al., 2011; Yang et al., 2012), the current study showed significantly greater amygdala responses to faces relative to objects. However, in contrast to Yang et al. (2012), but in line with the findings of Coker-Appiah et al. (2013) and Cao et al. (2014), there was a main effect of emotion, but not a stimulus-type by emotion interaction. As such, the current data indicate that animate and inanimate threatening stimuli, when matched for arousal and valence, generate comparable amygdala activity.

The effects of exposure were more complicated than we had anticipated. They were seen within dmFC/ACC, motor, parietal cortex and visual cortex replicating previous work (Wright et al., 2001; Phan et al., 2003; Yamaguchi et al., 2004; Summerfield et al., 2008; Weigelt et al., 2008). However, they were not seen within the amygdala. Moreover, several regions such as visual cortex, precuneus, culmen, and thalamus as well as the amygdala, showed interactions between emotion and exposure or emotion and congruence and exposure. With respect to the emotion-by-exposure interactions seen within bilateral visual cortex and left culmen, this primarily reflected greater habituation for neutral relative to negative stimuli; i.e., the reduction in activity on exposure 2 was particularly marked for neutral stimuli (Figure 3). However, for the regions showing interactions between congruence and exposure (and emotion), this reflected instead a greater differentiation between incongruent and congruent trials on exposure 2 relative to exposure 1, particularly if they involved negative stimuli. Habituation reflects a basic form of learning where there is a decrease in response to a stimulus following repeated exposure *with no meaningful consequence* (Rankin et al., 2009). We suggest that the absence of habituation seen in several areas, particularly for negative incongruent trials, reflects that for these trials the information had *meaningful consequence*. Future work will investigate this issue more deeply.

Previous work has reported that objects are associated with greater activity within the medial portion of the fusiform gyrus and middle temporal gyrus (Beauchamp et al., 2002; Mahon et al., 2007; Gobbini et al., 2011), while faces and other animate stimuli are associated within greater activity within FFA (Kanwisher and Yovel, 2006; Nguyen et al., 2013) and posterior STS (Beauchamp et al., 2002, 2003; Chao et al., 2002; Beauchamp and Martin, 2007; Martin, 2007; Gobbini et al., 2011). It is argued that object concepts belonging to different categories are represented in partially distinct, sensory- and motor property–based neural networks (Caramazza and Shelton, 1998; Martin, 2007). The current results were consistent with this previous research. Medial fusiform gyrus showed greater responses to objects relative to faces while STS showed greater responses to faces relative to objects. Interestingly, no finding was observed in the FFA. We suggest that the sub-threshold finding in STS and the lack of a finding in FFA may reflect parameters of our task where participants had to respond to cues devoid of animacy information.

Our predictions regarding category specific modulation by emotion were only partially supported. Given the direct feedback projections from the amygdala to visual processing areas, including temporal cortex (Pessoa et al., 2002; Mitchell et al., 2007), we had hypothesized that emotional modulation would only occur in medial fusiform cortex for inanimate objects and only in lateral fusiform cortex (including FFA and STS) for faces. However, no emotion-by-object type interactions within temporal cortex were observed. Even at a lenient threshold (*p* = 0.005, *k* > 10) no significant emotion-by-object type or emotion-by-object type-by-congruence interactions were observed. There was, though, differential connectivity by stimulus category as a function of emotion between bilateral amygdala and left medial fusiform gyrus. Significantly greater functional connectivity between left and right amygdala and left medial fusiform gyrus was observed for threatening objects relative to neutral objects, but not between fearful faces relative to neutral faces. This would suggest a degree of integrated functioning between the amygdala and the region of medial fusiform gyrus implicated in processing objects with respect to the emotional significance of objects. However, we found no evidence of a comparable process within lateral fusiform gyrus or STS for face stimuli (fearful relative to neutral). We again suggest that partial findings may reflect parameters of our task where participants had to respond to cues devoid of animacy information. This process is something we will investigate in future work.

Three caveats should be noted with respect to the current data. First, previous work with dot probe tasks has reported that participants respond more quickly to congruent relative to incongruent trials (Corbetta and Shulman, 2002). Moreover, previous work has reported that inferior frontal gyrus (iFG), medial frontal gyrus (mFG), and temporal-parietal junction (TPJ) show greater activation in incongruent relative to congruent trials (Corbetta and Shulman, 2002). In the current study, no regions survived corrections for multiple comparisons for the main effect of congruence in the whole brain fMRI analysis. However, it should be noted that an emotion-by-congruence-by-exposure interaction was observed in iFG. The expected increase in activation to incongruent relative to congruent stimuli was observed here, albeit only for negative stimuli during second exposures. A congruence effect restricted to negative stimuli was also observed in the amygdala. It is interesting to note here though that while the congruence effect was present for neutral stimuli it was not significant for emotional stimuli. This is consistent with a body of studies where the congruence effect in dot probe tasks, in at least healthy participants, is abolished if the stimuli are threatening (e.g., Waters et al., 2010). In short, we believe the weak response latency and BOLD response congruence effect seen here may reflect the use of emotional primes. Second, the current study used only faces as animate stimuli. The current results therefore may not generalize to other animate stimuli, such as animals. Third, it is possible that by selecting faces and objects matched for arousal and/or valence, we may have artificially removed regions displaying an emotion-by-animacy interaction; i.e., there may be a greater differentiation in participant judgments and BOLD response between emotional and neutral faces relative to emotional and neutral objects and by matching for judgment (faces vs. objects), we effectively matched for BOLD response. We cannot discount this possibility. We can only be confident, on the basis of the current data, that there is no interaction for matched stimuli.

In summary, the current results support suggestions that the amygdala is both responsive to animate as well as emotional stimuli. Additionally, these data suggest that the interaction between the various functions of the amygdala may need to be considered simultaneously to fully understand how they interact. Moreover, they suggest category-specific modulation of medial fusiform cortex as a function of emotion. In our future work, we aim to determine whether psychiatric conditions associated with amygdala dysfunction, particularly Conduct Disorder (Blair, 2013), PTSD (Admon et al., 2013), and mood and anxiety disorders (Damsa et al., 2009; Kerestes et al., 2013), show impairment in both the amygdala's responsiveness to emotional and face/animacy information.

### Conflict of interest statement

The authors declare that the research was conducted in the absence of any commercial or financial relationships that could be construed as a potential conflict of interest.
